# Business resilience skills for SMEs

**DOI:** 10.1186/s13731-023-00304-0

**Published:** 2023-06-01

**Authors:** Panagiotis Kotsios

**Affiliations:** Hellenic Open University, Patra, Greece

**Keywords:** Resilience, Skills, Survey, Poland, Greece

## Abstract

The goal of this research was to investigate the skills and values that can be related to building resilient Small and Medium Enterprises. Primary data on the topic were collected through survey research in a sample of 266 Greek and Polish business owners and managers during the summer of 2020. According to the replies, the Personal characteristics and Values category had the highest importance levels, but their adequacy levels were high as well. Especially Reliability, Integrity and Work ethics have been pointed out as vital for the long-term viability of a business while facing crises situations. The largest mismatch between importance and adequacy, by order of importance, were Communication, Risk identification and assessment, Financial Management, Planning and organisation and Customer-orientation, and these may constitute priority areas for inclusion in business training programs.

## Introduction

The COVID-19 pandemic has brought into the surface the need of businesses to adapt to unexpected shocks from the external environment and fast changing market conditions and regulations. This adaptation requires preparedness, flexibility, determination and a large set of other skills and values that will assist business owners survive through crises periods. Meanwhile, governments are required to provide adequate support to businesses that are most affected, while educators and counsellors are called to provide the necessary training and guidance. Apart from the “hard”, technical, sector-specific skills and knowledge that business owners should possess to continue their daily operations during the crisis period, “soft” skills can also play a crucial role in business continuity.

Soft skills were defined by Weber et al., (2011, p. 98). as “*interpersonal, human, people, or behavioral skills needed to apply technical skills and knowledge in the workplace”*, while Moss and Tilly (1996) defined them as “*skills, abilities, and traits that pertain to personality, attitude and behavior rather than to formal or technical knowledge”* (p. 256). On the context of the European Union, increased emphasis has been placed on the topic of skill development. Projects like ESCO (the European Skills, Competences, Qualifications and Occupations) and EntreComp (the Entrepreneurship Competence Framework), funding programs like Erasmus + and actions like the European Skills Agenda and the new Pact for Skills are directly aimed at skill development within the Union. These actions aim to involve a wide range of actors in the process, such as private companies, sectoral organisations, chambers of commerce, national and regional authorities, social partners, education and training providers, workers and employment services. One of the aims of the skill development process is to assist entrepreneurs and Small and Medium Enterprises (SMEs)—which represent 99% of all businesses in the EU (European Commission, 2022)—to develop the resilience skills that are needed for facing unexpected and impactful conditions. In relation to this goal, the current research aimed to collect and analyse primary information about the soft skills and values that can be directly related to crisis management and SME resilience.

## Business resilience

Τhe concept of resilience originally emerged in ecological literature (Holling, 1973), but is currently being used in several other scientific fields, like engineering (Hollnagel et al, 2006), psychology (Bonanno, 2004), sociology (Adger, 2000), disaster management (Manyena, 2006) and business administration (Sutcliffe & Vogus, 2003). Walker et al. (2004) mention that from a general social–ecological perspective, resilience can be defined as “*the capacity of a system to absorb disturbance and reorganise while undergoing change so as to still retain essentially the same function, structure, identity, and feedbacks*” (p. 4). Based on the same concept, the term resilience is increasingly met in entrepreneurship and business literature.

Sutcliffe and Vogus (2003) noted that the concept of resilience, whether used in the context of individuals or organisations, is generally founded on the notion of performing well, combined with the idea of difficult circumstances threatening to jeopardize such performance. They refer to the ability to “*preserve functioning*” despite the presence of internal or external adversities and recover from untoward events (p. 96). Ortiz de Mandojana and Bansal (2016, p. 1615) refer to business resilience as having the ability to “*anticipate, avoid, and adjust to shocks in their environment*”. Much of resilience literature reflects on the above-mentioned ideas. Burnard and Bhamra (2011), as well as Sheffi (2007), place emphasis on the detection and activation of appropriate organisational responses to significant external events. Lengnick-Hall et al. (2011) mention that organizational resilience has two differing perspectives: one related to the ability to survive from adversities and another one related to the development of new capabilities and the exploration of new opportunities. Hiles (2014) links the terms business recovery, continuity and resilience, by mentioning that “*the concept of business recovery—having a failure and recovering from it—has been succeeded by business continuity (BC)—being able to continue operations without hiatus in the event of disruption to any part of the operation. From there, it is a short step to inbuild resiliency*” (p.14). Beech et al (2019), as well as Wishart (2018), provide analytical reviews of the different approaches, perspectives and interpretations of resilience that have been adopted by various authors. Linnenluecke (2017) also offers an extensive review of influential publications on the topic.

Business resilience can be challenged by several, both internal and external, risk factors. Internal factors are related to core business aspects, such as the business model and value proposition, location, management aspects and practices, relationships between owners, employees’ safety, access to resources etc. External risk factors may be related to natural risks (e.g., floods, wildfires and pandemics), geopolitical risks (e.g., wars and acts of terrorism), economic risks (e.g., recessions and crises), technological risks (e.g., system breakdowns and cyberattacks), corporate risks (e.g., fraud and legal claims) and miscellaneous risks (e.g., oil spillovers and plane crashes) (Hiles, 2014). Moreover, as we live in an increasingly economically and technologically interdependent world, threats now come not only from a local, but also from a global scale. Business resilience is related to the ability of businesses to adapt and survive through such unexpected events. Having examined definitions of business resilience and some types of risks that new and existing business may face, it is important to mention the factors that can enhance business resilience. These factors can be divided at micro and macro level: micro-level factors are related to entrepreneurs and their businesses, while macro level factors are related to governments and the general economic and social environment.

### Micro level

Korber and McNaughton (2018) in their research point out the fact that a large part of entrepreneurial resilience literature supports the view that inherent characteristics like psychological traits and organizational characteristics of individuals or firms can increase resilience, which in turn enhances the ability of businesses to overcome disruptions. Many publications conceptualize entrepreneurial resilience as an amalgam of individual traits or qualities like flexibility, motivation, perseverance, optimism, self-efficacy and hope (De Vries & Shields, 2006; Hmieleski et al., 2015). Another key personal characteristic is that entrepreneurs tend to take responsibility for their own future and usually attribute success as well as failure on themselves, and this may be an important underlying factor for entrepreneurial resilience and drive (Hedner et al, 2011).

However, seeing resilience only as a result of personal attribute is risky, as it may limit our understanding of the related processes and willingness to provide adequate training and support. Davidsson et al. (2001) argued that in today’s research on entrepreneurship, the focus is shifting more towards the behavioural and cognitive aspects of the field, rather than the personality characteristics. Branicki et al. (2018) found a strong relationship between individual, entrepreneurial and SME resilience, noting that these concepts in many cases complement one another. Other papers stress the role of social capital (e.g., trust-based networks and support from family or friends) in assisting entrepreneurs to face crises situations (e.g.Bowey & Easton, 2007; Chrisman et al, 2011; Danes, 2013; Torres et al, 2019). Hedner et al (2011) mention that the concept of resilience is closely related to relationships that provide care and support, create trust and offer encouragement, both within and outside the family. Lee and Wang (2017) mention that a supportive family can be both a source of finances and psychological support.

For Teixeira and Werther (2013) resilience is evident in the way that organisations respond to changes—firms that anticipate events and changes and act to mitigate them in advance, and that do so repeatedly, are truly resilient. In this way, resilience is seen as closely related to competitive advantage, and building a resilient organisation is presented as a strategic imperative. Beech et al. (2019) supported the view that a key element of resilience is the coherent and rigorous nature of an organisations strategic thinking and decision-making capability within its leadership team. Hamel and Valikangas (2003) considered as important ingredients of resilience the capacity to retrieve and process information faster than competitors, embrace change and take adequate strategic action. Sheffi and Rice (2005) highlighted the relationship between resilience and organizational flexibility, mentioning that: “*as companies move to build flexibility in order to respond to demand and supply volatility, they are also building in resilience and *vice versa” (p.48). Smallborne et al. (2012), in their research for UK and New Zealand SMEs confirmed the importance of flexibility and adaptability in business resilience. Sabatino (2016) found that the most resilient enterprises are those that simplify their business structure and focus on their core competences. Granig and Hilgarter (2020) identified as key drivers for resilience organisational values and characteristics such as commitment, trust, empowerment, communication, leadership that provides a clear strategic direction and effective proactive risk management techniques.

Buliga et al (2016) in their study highlighted the role of business model innovation, considering it an as an integral part of organizational response and a constitutive element of resilience. Juettner and Maklan (2011) examined supply chain resilience in the global financial crisis, and concluded that four resilience capabilities—flexibility, reaction speed, access to timely information, and collaborations among supply chain members—can avoid or limit the impacts of adverse events. Sitkin (1992) highlighted the value of organizational learning from failure as an integral part of resilience. Moreover, Sincorá et al. (2018) highlighted the contribution of business analytics leveraging resilience in organizational processes. Hirt et al. (2019) also supported that digital and analytics-driven productivity improvements may be an important alternative to conventional cost cuts, and that accelerating digitization has widened the gap in capabilities and performance between digital leaders and laggards—a gap that is likely to grow during any downturn.

### Macro level

The extent of entrepreneurial resilience may not only be dependent on personal characteristics, or organizational strategies and social capital, but also on structural external factors that affect entrepreneurship and business survival in total. Korber & McNaughton (2018) in their research explored various macro-level factors that can enhance entrepreneurial resilience at the organizational or individual level. Examples include a competitive business environment (Biswas & Baptista, 2012), financial support through microfinance institutions (Ngoasong & Kimbu, 2016), training and mentoring programs for entrepreneurs (Ghosh & Rajaram, 2015; St-Jean & Audet, 2012) and better institutional structures (Sobel, 2008). Hedner et al (2011) supported the view that the social attitude towards business failure also plays a role. For example, the acceptance of failure is higher in the United States and considered as an experience for future success, while in Japan and Europe entrepreneurial failure may create a social stigma (European Commission, 2003; Vaillant & Lafuente, 2007). Bishop and Shilcof (2017, p. 215) in their research emphasized the role of entrepreneurial culture, flexibility, innovation, favourable industrial structures and diverse knowledge bases that assist some regions in fostering more resilient enterprises than others. On the contrary, factors that may prohibit resilient and productive entrepreneurship may be related to corruption (Aidis & Mickiewicz, 2006; Barkhatova, 2000) and burdensome business regulations (Djankov et al., 2002).

A final aspect of the business resilience literature is highlighted by Wishart (2018), who points out the limited focus that has been placed on the specific context of SMEs. Even though the contribution of SMEs in national economies is very important (for example, in the EU they account for 99% of total enterprises, two thirds of employment and 57% of value added), most of the focus up to date has been on large enterprises. Ates and Bitici (2011) point out the fact that are significant differences in the way that SMEs run their businesses and a different approach for resiliency is required. For example, Petrakis and Kostis (2015) researched the role of knowledge and interpersonal trust on SMEs, and found that knowledge positively affects the number of SMEs, which in turn positively affects interpersonal trust.

In conclusion, business resilience refers to the ability of a business or organization to absorb, adjust and continue its operations through time, and especially after the occurrence of impactful events. The events may be of a natural, economic, political, legal, technological or interorganizational origin, and if not addressed accordingly, may seriously undermine the sustainability of a business. On this context, business research is placing increased emphasis on the factors that determine business resilience and ability to maintain operation. These factors may be related, to a certain extent, with entrepreneurs’ personal characteristics that enable them to endure and face crises, and to another extent they are related to skills that can be developed through education and training. Other factors are related to the competitive strategies that businesses develop, as well as with social capital and general support from friends and family. Moreover, there also macro factors that affect business resilience, and they have to do to with the general support from the business environment in terms of financing and mentoring, the business culture of an area and the attitude towards failure.

On this context of micro and macro factors that can affect business resilience, the focus of the current research was on the topic of the soft skills. The main goal of the research was to investigate the soft skills that can be related to business resilience, by asking business owners from two European countries, Greece and Poland, their opinions on the matter.^1^ The methodology that was followed for the data collection is described below.

## Methodology

Primary data on the topic of soft skills that are related to SME resilience was collected through a survey on active Polish and Greek entrepreneurs, and the survey was based on a structured questionnaire.

*Questionnaire design* The first step in the research was to construct the survey’s questionnaire. The survey started by providing information about the researchers, the context and the goals of the research and by asking for the participants’ consent for taking part in the survey and for data processing. It is important to mention that participation in the research was voluntary and anonymous. The body of the questionnaire had four parts:

- The **1st part** was asking for basic demographic information about the business (*Name, Legal Form, City, Country, Main sector of activity, Year of establishment, Number of employees*) and the respondent (*Role in the business, Age group, Sex, Marital Status, Number of children and Higher completed educational level*).
- In the **2nd part** the respondents were asked to answer on a scale from 0 to 10 in 5 generic questions on the following topics: *1. The importance of business training, 2. The frequency that they receive business training, 3. The effects from COVID-19 crisis to their business, 4. The effects from other crises in the past (plus and open-ended question about the types of crises that affected them the most) and 5. Their overall business resiliency.*
- In the **3rd part** the participants were asked to grade on a scale from 0 to 10 the importance and personal adequacy of 36 skills that were chosen by the collaborating partners^2^ as important for the survival of a business during turbulent periods of time. For the construction of the skills’ list the researchers were based on the following sources of information:
  - The literature review on business resilience.
  - The European Commission’s Entrepreneurship Competence Framework EntreComp (Bacigalupo et al., 2016).
  - IME GSEVEE’s Typology of “skills mix” for their analysis and monitoring in the selected professions (Litzeris, 2019).
  - The European Commission’s European Classification of Skills/Competences, Qualifications and Occupations—ESCO (2020)
  - CEDEFOP’s European Skills Index (2020)
  - The collaborating partners experience in mentoring and advising entrepreneurs.

After three rounds of intellectual conversation between the partners, the final version of the skills’ list was constructed, and it presented in Table 1. It is vital to mention that the research focused only in soft skills and not sector-specific hard skills. The list includes 5 general soft skills categories and 36 specific skills and characteristics. The 5 skill categories are: (a) General skills, (b) Vocational/Professional skills, (c) Health and Safety (this category was included as an adaptation to the COVID19 outbreak) and finally (d) Risk Management. The list also included some important Personal characteristics, Attitudes and Values that are related to business continuity.

- The **4th and last part** of the questionnaire included 2 open-ended questions that aimed to cover possible gaps in the skills’ list. These were the following:
  - Which other skills do you consider important for the survival of your business?
  - What kind of business-related training, not presently available, would be useful for you?

**Table 1:** Skills’ list

Category	Subcategory	Soft skills
General skills (GS)	Cognitive of higher level	Goal Setting
		Ability for continuous learning
		Critical thinking
		Creativity
	Socio-emotional	Communication
		Empathy
	Systemic	Cooperation, Teamwork
		Adaptability (incl. Flexibility)
		Planning and organisation
		Negotiation
		Decision making
		Leadership
		Mobilising Resources
Vocational/professional skills (VPS)	General professional skills	Time management
		Customer-orientation
		Networking
	Digital skills	Digital Communication
		Data Security
	Data and information management	Data and information management and transformation
		Accessing, extracting and processing information
		Evaluation, analysis and synthesis of information and data
	Other professional skills	Financial and economic resources management
		Teaching, supporting and guiding other people
Health and safety (HS)	Health and safety	Providing for health and safety at work
		Ensuring public health and consumer protection
Characteristics, attitudes and values (CAV)	Personal characteristics/Attitudes—Behaviours	Reliability
		Initiative and taking action
		Self-confidence
		Perseverance
	Values	Integrity
		Work ethics
Resilience strategy (RS)	Risk management	Risk identification and assessment
		Resilience planning
		Impact analysis
		Recovery Strategy, Communication and Coordination
		Seeking advice–Receptiveness–Openness

*Methods of delivery* The questionnaire was available both in paper and digital form (as a word and pdf file), and also as an online survey in the LimeSurvey platform. The questionnaire was translated in 3 languages: English, Polish and Greek.

*Target audience* The questionnaire was addressed to active owners and managers of SMEs in Poland and Greece.

*Data collection methods* Data were collected through the online questionnaire, telephone interviews and live interviews in the premises of Family Business Foundation in Warsaw, Poland and the branches of the Hellenic Confederation of Professionals, Craftsmen and Merchants (IME GSEVEE) in Herakleion, Thessaloniki and Ioannina, Greece.

*Data collection period* The questionnaire design stage started on the 20th of May and was concluded on the 5th of June 2020. From the 8th to the 12th of June there was a pilot testing period, to get initial feedback and perform corrections. The research was officially launched in the 15th of June 2020. The first deadline for collecting the necessary answers was the 8th of July, which was expanded to the 20th of July. The goal was to get at least 100 replies from each country.

## Results and discussion

Following is the analysis of the SME owners and managers’ replies. It is important to mention that answering each question was optional. The replies gathered were 266 in total, 165 from Greece and 101 from Poland.^3^

The first question was asking about the enterprises’ year of establishment and the frequency of replies is presented in Table 2. Most of them were established between 1980 and 2000 (36%), meaning that they had between 20 and 40 years of active business experience. It is also worth noting that 28% of the enterprises were established during the last decade. The oldest one was established in 1914 and the newest one in 2020.

**Table 2:** Year of establishment

Year of establishment	*n*	%
1900–1950	2	0.79
1951–1980	12	4.76
1980–2000	92	36.51
2001–2010	75	29.76
2011–2020	71	28.17
Total	252	100.00

Table 3 presents the enterprises’ number of employees. Most of them can be characterized as micro enterprises, having from 0 to 9 employees (75%).^4^ The smallest value was 0, meaning that that they did not have any employees at all, and the largest one was 350.

**Table 3:** Number of employees

Number of employees	*n*	%
0–9 Micro	187	75.40
10–49 Small	38	15.32
50–249 Medium	20	8.06
250 + Large	3	1.21
Total	248	100.00

The respondents’ age groups are presented in Table 4. Most of them were in the age group between 36 and 45 (39%), and the second group in frequency was that between 46 and 55 (28%).

**Table 4:** Respondents’ age groups

Age group	*n*	%
18–25	3	1.16
26–35	29	11.20
36–45	102	39.38
46–55	73	28.19
56–65	43	16.60
65 +	9	3.47
Total	259	100.00

Regarding the respondents’ sex, these results are presented in Table 5. Most of the respondents were male, at a percentage of 65%.

**Table 5:** Sex

Sex	*n*	%
Female	92	34.98
Male	171	65.02
Total	263	100,00

The marital status is presented in Table 6, with most of the respondents being married to a percentage of 83%.

**Table 6:** Marital status

Marital status	*n*	%
Married	214	83.27
Single	27	10.51
Divorced	12	4.67
Separated	1	0.39
Widowed	3	1.17
Total	257	100.00

The number of children is presented in Table 7, with 83% of the respondents having children.

**Table 7:** Number of Children

No of children	*n*	%
0	41	16.33
1	65	25.90
2	100	39.84
3	35	13.94
4	9	3.59
5	1	0.40
Total	251	100.00

Finally, Table 8 presents the participants’ highest completed educational level, with 37% of them having a Master’s degree, 26% having a Bachelor degree and 27% having completed secondary education. The total percentage with completed higher education was 68% (Tertiary, Master’s and Doctorate).

**Table 8:** Highest educational level completed

Educational level	*n*	%
Primary	6	2.30
Secondary	71	27.20
Tertiary	70	26.82
Master’s	99	37.93
Doctorate	7	2.68
Other	8	3.07
Total	261	100.00

The second part of the questionnaire included some generic questions about business training, crisis and resilience. The questions and average scores are presented in Table 9. The respondents considered business training as very important and they declared that they receive training, but not very often. In addition, according to the replies, they have been affected by the COVID-19 crisis at a considerable level, and they have been also affected by other crises in the past, but to a smaller degree. The 4th question also had an open-end part, which was asking about the type of crises that had affected them most in the past. The answers received were 243, and most of the respondents mentioned the economic crisis at a percentage of about 80%. Some other answers included technological, political and natural crises, as well as inner-business relationships.

**Table 9:** Training and resilience questions

Business training importance *[On a scale of 0 (not at all) to 10 (very much), to what degree do you think that business training and skill development can help your business stay active in the market and grow?]*	8.45 (SD 1.76)
Business training frequency *[On a scale of 0 (never) to 10 (very frequently), how often do you receive business-related training?]*	5.36 (SD 2.69)
Effects from COVI19 crisis *[On a scale of 0 (not at all) to 10 (very much), at what degree do think that your business has been (or will be) negatively affected by the COVID-19 crisis?]*	6.85 (SD 2.79)
Effects from other crises *[On a scale of 0 (not at all) to 10 (very much), at what degree has your business been influenced by other crises in the past (apart from COVID-19)?]*	5.49 (SD 2.99)
Business resiliency *[On a scale of 0 (not at all) to 10 (very much), how resilient do you consider you enterprise as a whole?]*	7.00 (SD 2.69)

The third part of the questionnaire was asking participants to assess the importance and personal adequacy of the skills in the list. Table 10 presents the skills’ average importance, adequacy and mismatch, by order of importance.

**Table 10:** Skills average importance, adequacy and mismatch by order of importance

No	Cat	Skill	Importance	Adequacy	Mismatch
1	CAV	Reliability	9.50	8.82	0.68
2	CAV	Integrity	9.47	9.23	0.23
3	CAV	Work ethics	9.39	9.25	0.14
4	GS	Communication	9.22	7.70	**1.52**
5	CAV	Perseverance	9.10	8.30	0.80
6	CAV	Initiative and taking action	9.03	8.17	0.87
7	GS	Decision making	9.03	7.86	1.16
8	RS	Risk identification and assessment	8.98	7.53	**1.45**
9	VPS	Financial and economic resources management	8.95	7.56	**1.40**
10	GS	Planning and organisation	8.95	7.47	**1.48**
11	GS	Adaptability (incl. Flexibility)	8.94	7.72	1.21
12	VPS	Customer-orientation	8.91	7.43	**1.48**
13	GS	Leadership	8.80	7.61	1.19
14	CAV	Self-confidence	8.79	7.95	0.85
15	VPS	Networking	8.78	7.09	**1.69**
16	GS	Cooperation. Teamwork	8.73	7.66	1.07
17	RS	Recovery Strategy. Communication and Coordination	8.72	7.15	**1.57**
18	GS	Creativity	8.72	7.68	1.04
19	VPS	Digital Communication	8.70	7.27	1.43
20	VPS	Time management	8.70	7.04	**1.66**
21	GS	Mobilising Resources	8.69	7.22	**1.48**
22	RS	Seeking advice–Receptiveness–Openness	8.67	7.28	1.39
23	HS	Providing for health and safety at work	8.62	8.23	0.39
24	GS	Continuous learning	8.62	7.16	**1.46**
25	HS	Ensuring public health and consumer protection	8.59	8.18	0.41
26	VPS	Teaching. supporting and guiding other people	8.56	7.28	1.28
27	RS	Resilience planning	8.50	7.05	1.45
28	GS	Goal Setting	8.46	7.18	1.28
29	GS	Negotiation	8.41	7.16	1.24
30	RS	Impact analysis	8.40	7.00	1.40
31	VPS	Data Security	8.39	7.42	0.97
32	GS	Critical thinking	8.35	7.51	0.84
33	GS	Empathy	8.30	7.62	0.69
34	VPS	Evaluation. analysis and synthesis of information and data	8.29	7.00	1.28
35	VPS	Data and information management and transformation	8.00	6.93	1.07
36	VPS	Accessing. extracting and processing information	7.91	6.84	1.07
Min			7.91	6.84	0.14
Max			9.50	9.25	1.69
Range			1.58	2.41	1.55

The highest values are given in bold

From the analysis of the results, the following observations can be made:

- All the skills and characteristics in the list were evaluated as particularly important from the respondents, since all the average scores are high, ranging from 7.91 (min) to 9.50 (max).
- Surprisingly, the highest scores were in the Characteristics, Attitudes and Values category, and in particular in Reliability (9.50), Integrity (9.47) and Work ethics (9.39). Perseverance and Initiative and action taking had also very high scores (9.10 and 9.03, respectively). However, adequacy levels in these characteristics and values was high as well.
- The soft skills with the highest scores were Communication (9.22); Decision making (9.03); Risk identification and assessment (8.98); Financial management (8.95) and Planning and Organization (8.95).
- The respondents auto-assessed their adequacy in possessing these skills as high, with values ranging from 6.84 (min) to 9.25 (max). The lowest adequacies can be found in Accessing, extracting and processing information (6.84); Data and information management and transformation (6.93); Evaluation, analysis and synthesis of information and data (7.00); Impact analysis (7.00); and Time management (7.04).
- The largest mismatches, between importance and adequacy can be traced in Networking (1.69); Time management (1.66); Recovery strategy (1.57) and Communication (1.52). By order of importance, the skills with the largest mismatch were Communication (1.52); Risk identification and assessment (1.45); Financial Management (1.40); Planning and organisation (1.48); Customer-orientation (1.48); Networking (1.69); Recovery Strategy (1.57); Time management (1.66); Mobilising Resources (1.48) and Continuous learning (1.46).

The last part of the questionnaire included two open-end questions. The first on was asking about the skills the participants considered as most important for the survival of a business. The percentage of participants that replied was 52%. Some of the skills mentioned more frequently were related to business management, financial management, debt management, funding, risk and change management, new technologies, digital marketing, sales promotion and project management. The second question was asking about the type of business training they would like to receive. The percentage of participants that replied was 66%. Most frequent answers mentioned Financial management, Digital marketing, Sales promotion, Human resource selection and management, Project management, Crisis management, New technologies, Cost analysis, Labelling, Taxation, Health and safety and Legal requirements. Many answers also referred to sector-specific skills.

### Comparison between countries

To check the validity of the results and identify possible differences between countries, the replies from Poland (101) and Greece (165) were compared in Table 11. Significant differences can be noticed in the Effects from the COVID-19 crisis, with business owners and managers from Greece declaring a more negative effect that Polish ones by 2.40, and also a higher effect from other crises in the past (difference of 2.74). Polish respondents considered on average their businesses more resilient that Greek ones, by a difference of 0.95. Regarding the importance and adequacy of the skills, there are some differences in average scores between countries. In terms of importance, the largest differences are spotted in Critical thinking; Empathy and Ensuring public health and consumer protection. In terms of adequacy, the largest differences are in Critical thinking; Self Confidence; Leadership and Communication.

**Table 11:** Comparison between Poland and Greece

Category	Question	Poland	Greece	Var
		*n* = 101	*n* = 165	
		Average	Average	
Generic Questions	Business training importance	7.86	8.81	− 0.95
	Business training frequency	4.77	5.72	− 0.95
	Effects from COVID-19 crisis	5.36	7.76	− 2.40
	Effects from other crises	3.78	6.52	− 2.74
	Business resiliency	7.59	6.64	0.95
General Skills	Goal Setting Importance	8.34	8.53	− 0.19
	Goal Setting Adequacy	6.94	7.33	− 0.39
	Continuous learning Importance	8.62	8.62	0.00
	Continuous learning Adequacy	7.32	7.07	0.25
	Critical thinking Importance	7.58	8.82	− **1.25**
	Critical thinking Adequacy	6.79	7.95	− **1.16**
	Creativity Importance	8.51	8.85	− 0.34
	Creativity Adequacy	7.35	7.87	− 0.52
	Communication Importance	9.08	9.30	− 0.22
	Communication Adequacy	7.14	8.03	− **0.90**
	Empathy Importance	7.71	8.65	− **0.94**
	Empathy Adequacy	7.32	7.79	− 0.47
	Cooperation, Teamwork Importance	8.54	8.84	− 0.30
	Cooperation, Teamwork Adequacy	7.38	7.83	− 0.46
	Adaptability (incl. Flexibility) Importance	8.65	9.11	− 0.46
	Adaptability (incl. Flexibility) Adequacy	7.66	7.76	− 0.10
	Planning and organisation Importance	8.62	9.15	− 0.52
	Planning and organisation Adequacy	7.16	7.66	− 0.50
	Negotiation Importance	7.96	8.68	− 0.72
	Negotiation Adequacy	6.80	7.38	− 0.58
	Decision making Importance	8.96	9.07	− 0.11
	Decision making Adequacy	7.76	7.93	− 0.17
	Leadership Importance	8.47	8.99	− 0.52
	Leadership Adequacy	7.03	7.96	− **0.93**
	Mobilising Resources Importance	8.58	8.76	− 0.18
	Mobilising Resources Adequacy	7.12	7.28	− 0.16
Professional Skills	Time management Importance	8.22	8.99	− 0.77
	Time management Adequacy	6.61	7.30	− 0.69
	Customer-orientation Importance	8.56	9.12	− 0.56
	Customer-orientation Adequacy	7.24	7.53	− 0.29
	Networking Importance	8.26	9.09	− 0.83
	Networking Adequacy	6.72	7.30	− 0.59
	Digital Communication Importance	8.36	8.91	− 0.55
	Digital Communication Adequacy	7.05	7.40	− 0.34
	Data Security Importance	8.52	8.31	0.21
	Data Security Adequacy	7.52	7.37	0.15
	Data and information management and transformation Importance	7.91	8.06	− 0.16
	Data and information management and transformation Adequacy	6.77	7.03	− 0.26
	Accessing, extracting and processing information Importance	7.52	8.14	− 0.62
	Accessing, extracting and processing information Adequacy	6.51	7.03	− 0.52
	Evaluation, analysis and synthesis of information and data Importance	8.01	8.45	− 0.44
	Evaluation, analysis and synthesis of information and data Adequacy	6.62	7.23	− 0.60
	Financial and economic resources management Importance	8.98	8.94	0.04
	Financial and economic resources management Adequacy	7.69	7.48	0.21
	Teaching, supporting and guiding other people Importance	8.49	8.60	− 0.11
	Teaching, supporting and guiding other people Adequacy	7.12	7.38	− 0.26
Health and Safety	Providing for health and safety at work Importance	7.99	9.00	− 1.01
	Providing for health and safety at work Adequacy	8.32	8.17	0.15
	Ensuring public health and consumer protection Importance	7.91	8.99	− **1.09**
	Ensuring public health and consumer protection Adequacy	7.77	8.42	− 0.65
Characteristics, Attitudes and Values	Reliability Importance	9.43	9.53	− 0.10
	Reliability Adequacy	8.57	8.96	− 0.39
	Initiative and taking action Importance	8.95	9.09	− 0.14
	Initiative and taking action Adequacy	7.86	8.35	− 0.48
	Self-confidence Importance	8.27	9.10	− 0.84
	Self-confidence Adequacy	7.26	8.35	− **1.08**
	Perseverance Importance	9.10	9.10	0.00
	Perseverance Adequacy	8.04	8.45	− 0.41
	Integrity Importance	9.63	9.37	0.25
	Integrity Adequacy	9.51	9.07	0.43
	Work ethics Importance	9.47	9.34	0.13
	Work ethics Adequacy	9.20	9.29	− 0.09
Risk Management	Risk identification and assessment Importance	8.95	8.99	− 0.05
	Risk identification and assessment Adequacy	7.23	7.70	− 0.48
	Resilience planning Importance	8.10	8.74	− 0.64
	Resilience planning Adequacy	6.53	7.34	− 0.80
	Impact analysis Importance	7.97	8.65	− 0.68
	Impact analysis Adequacy	6.70	7.18	− 0.48
	Recovery Strategy, Communication and Coordination Importance	8.36	8.94	− 0.58
	Recovery Strategy, Communication and Coordination Adequacy	6.84	7.33	− 0.49
	Seeking advice–Receptiveness–Openness Importance	8.45	8.80	− 0.35
	Seeking advice–Receptiveness–Openness Adequacy	7.04	7.42	− 0.38
*n*		72	72	–
Mean (μ)		7.91	8.32	–
Standard deviation		0.83	0.73	–

The highest values are given in bold

The differences between the replies from the two countries was tested using the following hypotheses: Null Hypothesis Ho: ${\mu }_{1}$=${\mu }_{2}$, Alternative Hypothesis Ho: ${\mu }_{1}$≠${\mu }_{2}$. The method used to test the difference was a two-tailed *t* test at a significance level of 0.05 and 142 degrees of freedom. The critical value for this two-tailed test was t = 1.977. The calculated t value was -3.147 and the *p* value was 0.002 (< 0.05), so the null hypothesis was rejected. There was enough evidence to claim that population means between Poland and Greece were different at the 0.05 significance level.

### Clusters

Apart from the comparison in the responses from Poland and Greece, the sample was divided into smaller groups and compared with the total sample to examine possible differences in the replies. The three (3) groups used were the following (Table 12):

- **Group 1—business that were established prior to 1980**: This group represented those businesses that have proven their resilience by staying active for more than 40 years. Their number was 12.
- **Group 2—business owners and managers who have received higher education**: This group included those respondents that had completed a Bachelor, Masters or Doctorate degree. The goal was to examine if higher education creates differences in their replies from the total sample. Their number was 176.
- **Group 3—high business resilience**: This group included those respondents that had auto assessed their business’s resilience with a score of 8, 9 and 10. Their number was 109.

**Table 12:** Cluster analysis

Soft skills		Total	Year of Establ. < 1980		Higher education		High Bus. resilience	
		*n* = 266	*n* = 12		*n* = 176		*n* = 109	
		Aver.	Aver.	Var.	Aver.	Var.	Aver.	Var.
Generic Questions	Business training importance	8.45	7.92	0.53	8.57	− 0.12	8.34	0.11
	Business training frequency	5.36	5.42	− 0.06	5.62	− 0.26	5.56	− 0.20
	Effects from COVI19 crisis	6.85	7.17	− 0.32	6.66	0.19	5.86	**0.99**
	Effects from other crises	5.49	6.75	− **1.26**	5.34	0.16	5.01	0.48
	Business resiliency	7.81	7.17	0.64	7.16	0.65	8.67	− **0.86**
Generic Skills	Goal Setting Importance	8.96	8.42	0.54	8.51	0.46	8.58	0.38
	Goal Setting Adequacy	7.31	7.50	− 0.19	7.28	0.02	7.43	− 0.12
	Continuous learning Importance	8.73	9.00	− 0.27	8.60	0.13	8.83	− 0.10
	Continuous learning Adequacy	7.77	7.67	0.10	7.34	0.43	7.67	0.10
	Critical thinking Importance	7.73	8.75	− **1.02**	8.38	− 0.65	8.31	− 0.57
	Critical thinking Adequacy	6.46	8.33	− **1.87**	7.59	− **1.13**	7.71	− **1.25**
	Creativity Importance	8.88	8.83	0.05	8.72	0.16	8.71	0.17
	Creativity Adequacy	7.88	8.17	− 0.29	7.66	0.22	7.80	0.08
	Communication Importance	9.21	9.33	− 0.13	9.24	− 0.03	9.22	− 0.02
	Communication Adequacy	7.75	8.00	− 0.25	7.65	0.10	7.87	− 0.12
	Empathy Importance	8.08	8.08	0.00	8.32	− 0.23	8.31	− 0.23
	Empathy Adequacy	7.54	7.67	− 0.13	7.62	− 0.08	7.78	− 0.24
	Cooperation, Teamwork Importance	9.00	8.83	0.17	8.75	0.25	8.82	0.18
	Cooperation, Teamwork Adequacy	7.54	7.36	0.18	7.62	− 0.08	7.87	− 0.32
	Adaptability (incl. Flexibility) Importance	9.30	8.91	0.40	8.87	0.44	8.84	0.46
	Adaptability (incl. Flexibility) Adequacy	8.22	7.82	0.40	7.78	0.44	8.10	0.11
	Planning and organisation Importance	9.21	8.75	0.46	8.98	0.23	8.79	0.42
	Planning and organisation Adequacy	7.38	7.58	− 0.21	7.56	− 0.18	7.57	− 0.19
	Negotiation Importance	7.92	8.55	− 0.63	8.47	− 0.55	8.31	− 0.39
	Negotiation Adequacy	7.17	7.45	− 0.29	7.11	0.05	7.35	− 0.19
	Decision making Importance	8.83	9.17	− 0.33	9.06	− 0.22	9.08	− 0.25
	Decision making Adequacy	7.63	8.33	− 0.71	7.81	− 0.18	8.16	− 0.54
	Leadership Importance	8.21	9.08	− **0.88**	8.80	− 0.60	8.76	− 0.55
	Leadership Adequacy	7.38	7.67	− 0.29	7.60	− 0.23	7.60	− 0.23
	Mobilising Resources Importance	9.00	9.17	− 0.17	8.77	0.23	8.74	0.26
	Mobilising Resources Adequacy	7.00	7.42	− 0.42	7.26	− 0.26	7.52	− 0.52
Professional Skills	Time management Importance	8.50	8.18	0.32	8.66	− 0.16	8.67	− 0.17
	Time management Adequacy	6.79	6.55	0.25	7.00	− 0.21	7.43	− 0.64
	Customer-orientation Importance	8.74	9.10	− 0.36	8.78	− 0.04	8.83	− 0.09
	Customer-orientation Adequacy	7.43	8.40	− **0.97**	7.46	− 0.02	7.80	− 0.36
	Networking Importance	8.29	9.00	− 0.71	8.53	− 0.24	8.53	− 0.24
	Networking Adequacy	6.92	7.82	− **0.90**	7.04	− 0.12	7.28	− 0.36
	Digital Communication Importance	8.46	9.09	− 0.63	8.65	− 0.19	8.58	− 0.12
	Digital Communication Adequacy	7.29	7.09	0.20	7.37	− 0.08	7.66	− 0.37
	Data Security Importance	8.50	8.55	− 0.05	8.47	0.03	8.54	− 0.04
	Data Security Adequacy	7.67	7.27	0.39	7.51	0.16	7.67	− 0.01
	Data and information management Importance	7.75	7.50	0.25	8.13	− 0.38	8.08	− 0.33
	Data and information management Adequacy	6.92	6.50	0.42	7.06	− 0.15	7.12	− 0.20
	Accessing, extracting and processing information Importance	7.39	7.08	0.31	7.96	− 0.57	7.83	− 0.44
	Accessing, extracting and processing information Adequacy	6.52	6.08	0.44	6.88	− 0.35	6.87	− 0.35
	Evaluation, analysis and synthesis of information and data Importance	7.91	7.64	0.28	8.35	− 0.44	8.21	− 0.29
	Evaluation, analysis and synthesis of information and data Adequacy	6.52	6.55	− 0.02	7.14	− 0.62	7.11	− 0.59
	Financial and economic resources management Importance	9.09	8.75	0.34	8.94	0.15	9.03	0.06
	Financial and economic resources management Adequacy	7.30	7.83	− 0.53	7.61	− 0.30	7.92	− 0.62
	Teaching, supporting and guiding other peopleImportance	8.70	8.67	0.03	8.60	0.09	8.60	0.09
	Teaching, supporting and guiding other peopleAdequacy	7.35	7.25	0.10	7.42	− 0.07	7.46	− 0.11
Health and Safety	Providing for health and safety at work Importance	8.71	9.67	− **0.96**	8.49	0.21	8.19	0.52
	Providing for health and safety at work Adequacy	8.88	8.67	0.21	8.27	0.61	8.42	0.46
	Ensuring public health and consumer protection Importance	8.83	9.50	− 0.67	8.49	0.34	8.10	**0.72**
	Ensuring public health and consumer protection Adequacy	8.13	8.75	− 0.62	8.10	0.03	8.06	0.07
Characteristics, Attitudes and Values	Reliability Importance	9.33	9.17	0.17	9.48	− 0.14	9.54	− 0.21
	Reliability Adequacy	8.50	8.75	− 0.25	8.79	− 0.29	8.94	− 0.44
	Initiative and taking action Importance	9.00	8.58	0.42	9.01	− 0.01	9.05	− 0.05
	Initiative and taking action Adequacy	8.04	7.83	0.21	8.17	− 0.13	8.34	− 0.30
	Self-confidence Importance	8.00	8.67	− 0.67	8.76	− **0.76**	8.58	− 0.58
	Self-confidence Adequacy	7.29	8.17	− **0.87**	7.92	− 0.63	8.02	− **0.73**
	Perseverance Importance	9.21	9.00	0.21	9.16	0.05	8.92	0.29
	Perseverance Adequacy	8.04	8.08	− 0.04	8.28	− 0.24	8.47	− 0.43
	Integrity Importance	9.50	8.92	0.58	9.49	0.01	9.51	− 0.01
	Integrity Adequacy	9.54	9.17	0.38	9.24	0.30	9.51	0.03
	Work ethics Importance	9.46	9.18	0.28	9.42	0.04	9.39	0.07
	Work ethics Adequacy	9.21	9.33	− 0.13	9.28	− 0.08	9.43	− 0.22
Risk Management	Risk identification and assessment Importance	9.04	8.92	0.13	8.94	0.10	9.09	− 0.05
	Risk identification and assessment Adequacy	7.57	7.67	− 0.10	7.52	0.05	7.72	− 0.16
	Resilience planning Importance	8.32	8.83	− 0.52	8.37	− 0.05	8.45	− 0.13
	Resilience planning Adequacy	6.82	7.92	− 1.10	6.98	− 0.16	7.20	− 0.38
	Impact analysis Importance	8.27	8.50	− 0.23	8.35	− 0.08	8.22	0.05
	Impact analysis Adequacy	6.73	7.42	− 0.69	6.99	− 0.27	7.22	− 0.49
	Recovery Strategy, Communication and Coordination Importance	8.48	8.75	− 0.27	8.67	− 0.19	8.56	− 0.08
	Recovery Strategy, Communication and Coordination Adequacy	6.65	7.50	− 0.85	7.18	− 0.52	7.37	− 0.72
	Seeking advice–Receptiveness–Openness Importance	9.23	8.83	0.39	8.72	0.51	8.45	**0.78**
	Seeking advice–Receptiveness–Openness Adequacy	7.50	7.58	− 0.08	7.38	0.12	7.26	0.24

The highest values are given in bold

Based on the results of these groups of respondents, the following observations can be made:

- In **Group 1**—owners or managers of businesses with at least 40 years of operation—we can notice important variance in the Effect from other crises in the past—1.26 higher than the overall sample average. In terms of importance there are worth noting variances in Critical thinking (+ 1.02), Providing for health and safety at work (+ 0.96) and Leadership (+ 0.88). In terms of adequacy there are variances in Critical thinking (+ 1.87), Customer-orientation (+ 0.97), Networking (+ 0.90) and Self-confidence (+ 0.87).
- In **Group 2**, owners or managers with completed higher education, in terms of importance there is variance in Self-confidence (+ 0.76) and in terms of adequacy in Critical thinking (+ 1.13).
- In **Group 3**, owners or managers with auto assessed their business’s resilience with 8.9 or 10, there is an important variance in the Effect from the COVID19 crisis—0.99 lower than the overall sample. In terms of importance there is a variance in Seeking advice–Receptiveness–Openness (− 0.78) and Ensuring public health and consumer protection (− 0.72), and in terms of adequacy in Critical thinking (+ 1.25) and in Self Confidence (+ 0.73).

## Conclusions

Through the results of this survey research it is possible to draw a series of conclusions that can be useful both for active and future entrepreneurs, as well as for educators and policy makers. These conclusions are related to the soft skills that entrepreneurs should have in mind to build resilient SMEs, as well as the soft skills that educators and policy makers should include and cultivate in business training programs.

A first conclusion is that all the 36 soft skills and values included in the list were considered as important for business survival and continuity by active business owners and managers. There was no skill or value pointed out as unimportant from the replies of the sample. A second conclusion is that the Characteristics, Attitudes and Values category had the highest scores in terms of importance, but overall their adequacy level was high as well. Especially Reliability, Integrity and Work ethics have been pointed out as vital for the long-term viability of a business while facing crises situations. These values are frequently mentioned in business training programs, but their practical cultivation is a challenging task, and one that requires extensive theoretical and practical business experience. A third set of conclusions is related to specific soft skills: the most important soft skills were Communication; Decision making; Risk identification and assessment; Financial management; and Planning and Organization. The lowest adequacies were spotted in skills related to data management: accessing, extracting and processing information; Data and information management and transformation; Evaluation, analysis and synthesis of information and data. Low adequacies were recorded also in Impact analysis and Time management. By order of importance, the ten skills with the largest mismatch between importance and adequacy were Communication; Risk identification and assessment; Financial Management; Planning and organisation; Customer-orientation; Networking; Recovery Strategy; Time management; Mobilising Resources and Continuous learning, and these may constitute priority areas for inclusion in training programs.

Another conclusion is that there were significant differences in the effects the COVID-19 and other crises of the past between Polish and Greek enterprises. In regard to the value of the specific skills, there were some differences in the population means and especially, in terms of importance in Critical thinking; Empathy; and Ensuring public health and consumer protection, while in terms of adequacy in Critical thinking; Self Confidence; Leadership; and Communication. A final set of conclusions is related to the replies of specific clusters of respondents. The owners or managers of businesses with at least 40 years of operation placed increased emphasis in Critical thinking; Providing for health and safety at work; and Leadership and in terms of adequacy there were favourable variances in Critical thinking; Customer-orientation; Networking; and Self-confidence. The owners or managers with completed higher education had worth noting importance variance in Self-confidence and adequacy variance in Critical thinking. Finally, the owners or managers who auto assessed their business’s resilience as very high, had a lower Effect from the COVID-19 crisis. The current research and the generalizability of its results are limited by the sample size and the margin of error (± 6%); however, a future research on a larger sample may shed further light on the topic of soft skills and SME resilience.

## Data Availability

Anonymized data are available upon request.

## Footnotes

1 The research was executed in the framework of the ERASMUS + project «*Resilience and rescue skills for SMEs, strengthening Early Warning Europe*», and the target group selection was related with the deliverables of the project.

2 The collaborating partners included representatives from the Business Development Centre of Central Denmark, the Cecot Innovation Foundation from Spain, the Family Business Foundation from Poland, the Athens Chamber of Tradesmen and the Hellenic Confederation of Professionals, Craftsmen and Merchants (IME GSEVEE) from Greece, the Athens Chamber of Tradesmen in Greece and the JAMK University of Applied Sciences from Finland.

3 *The total business population in Poland in 2018 was 1,732,623 (EC, *2019 SBA Fact Sheet*) and in Greece 1,195,299 (author’s calculations based on open data from the *Hellenic Independent Authority on Public Revenues*), so the total business population in these two countries was 2,927,922. With a sample of 266 from these two countries, the margin of error is 6%, meaning that there is a 95% chance that the real values are within* ± *6% of the measured/surveyed values.*

4 Enterprises’ sizes are defined by EU recommendation 2003/361.
